# Self-Rated Recovery and Mood Before and After Resistance Training and Muscle Microcurrent Application

**DOI:** 10.3389/fpsyg.2022.836695

**Published:** 2022-04-14

**Authors:** Bernd A. C. Stößlein, Kim P. C. Kuypers

**Affiliations:** Department of Neuropsychology and Psychopharmacology, Faculty of Psychology and Neuroscience, Maastricht University, Maastricht, Netherlands

**Keywords:** frequency-specific microcurrent, microstimulation, deadlifts, resistance training, self-rated recovery, mood

## Abstract

**Background:**

Resistance training (RT) can offer beneficial physiological and psychological effects. The regular continuation of this exercise can be accomplished by improving the recovery and mood after a workout. Frequency-specific microcurrent (microstimulation) might offer a solution here as it has been shown to improve physical injuries, mood state, and sleep. However, knowledge is lacking about the impact of microstimulation after RT on said parameters. The present study aimed to test the effects of RT and muscle-microstimulation on mood and physical recovery in healthy men after performing conventional deadlifts, which is a type of RT.

**Methods:**

The study was conducted according to a single-blind, randomized, placebo-controlled, and two-way crossover study. Twenty participants naïve to microstimulation (MS) engaged in RT twice on separate days. They were randomized to receive MS on 1 day and no microstimulation (Sham-MS) on another day. Before and after the workout and after their treatment (MS or Sham-MS), participants self-rated their mood state and mental and physical exhaustion levels.

**Results:**

Findings showed that MS increased the self-ratings of well-rested and sociable and, most importantly, reduced the feeling of exercise-induced exhaustion. There were no MS effects on ratings of feeling sad, happy, or exhausted, although the workout, independent of MS, negatively influenced the level of exhaustion.

**Conclusion:**

The combination of enhanced sociableness, reduced fatigue, and exercise-induced exhaustion after a workout, followed by microstimulation, has important implications for professional sporters and nonprofessionals who try to get the best result after a workout. Future studies using a double-blind approach including different types of exercises, different durations of programs, and both sexes can shed more light on the full potential of microstimulation after a workout on mood state and exercise-induced exhaustion.

## Introduction

Resistance training (RT) is a popular anaerobic physical activity among professionals and nonprofessionals. It can have beneficial effects across all ages because of its positive effects on muscle mass and strength, and bone mineral density (Yeung, 1996; Westcott, 2012; Mandolesi et al., 2018). The effects on psychological (mood) and physiological processes are, however, intensity-dependent (Westcott, 2012); with low-to-moderate intensity RT (at a smaller than 70% one-repetition maximum), anxiolytic effects in diverse populations are, for example, produced (Strickland and Smith, 2014).

Studies in aerobic exercise have shown that exercise adherence and the chance people will engage in such an activity in the future is higher when the person feels good doing the activity (Williams et al., 2008; Schneider et al., 2009; Rhodes and Kates, 2015). Improved or faster recovery after the exercise to reduce muscle soreness and hence improve the positive feeling connected to the exercise might contribute to the continuation of a specific exercise in the future and eventually contribute to overall better health (Chao et al., 2000; Williams et al., 2008; Schneider et al., 2009; Rhodes and Kates, 2015).

Frequency-Specific Microcurrent (microstimulation, MS) has been shown to contribute to muscle recovery (Cheng et al., 1982; Ahmed et al., 2012; Sharp et al., 2019). Microstimulation is the transcutaneous delivery of low amperage Direct Current (DC) and/or Alternating Current (AC) *via* adhesive electrodes with adjustable frequencies in the microampere (μA) range (Naclerio et al., 2019). Preclinical research in rats has demonstrated that the amperage of the current determines the biological effects (Cheng et al., 1982), i.e., the current has to be small enough to create intercellular modulations, like increased protein synthesis and elevated adenosine triphosphate (ATP) levels (Cheng et al., 1982), but not exceeding a certain amperage (e.g., above 1,000 μA) which leads to a decrease in ATP production (Cheng et al., 1982; McMakin, 2010).

Furthermore, preclinical and clinical work has shown that MS results in tissue repair on cellular levels and advancement in, for example, tendon healing (Ahmed et al., 2012; Sharp et al., 2019). Recently a study showed that MS speeds up physiological recovery after constant-load cycling (Piras et al., 2021). Additionally, the longer-lasting duration of positive effects of MS has also been demonstrated previously; A 20 min MS of healthy volunteers’ hamstring protected from pronounced self-rated delayed onset muscle soreness (DOMS) 24 h after exercises (sets of seated leg curls). This effect extended to 72 h after the exercises and the MS, compared to the sham-stimulation after exercises (Curtis et al., 2010). Of note, DOMS increased independently of treatment, i.e., with or without MS; however, it was lower in the MS condition than in the sham condition. The DOMS ratings returned to baseline 72 h post-exercise and MS, while still elevated in the sham condition (Curtis et al., 2010). When applied daily after upper and lower body exercises and at non-training days, 3 h of MS led to a greater pennation angle than sham-stimulation, reducing DOMS 12, 24, and 48 post-exercise (Naclerio et al., 2019).

Besides physical advantages, MS has beneficial effects on psychological parameters, like mood and arousal levels. For example, Cheung et al. (2015) demonstrated that MS at the Shenmen acupoint (HT7), located at the wrist crease of the radial side of the flexor carpi ulnaris tendon, led to a change in brain waves as determined with EEG compared with sham treatment. These activation patterns were previously related to mood-enhancement and decreased arousal levels or higher sleepiness; therefore, findings suggest that MS can affect these psychological processes (Cheung et al., 2015).

The present study was designed to address whether RT combined with microstimulation affects post-exercise recovery and positive mood. Based on the existing literature, it is hypothesized that physical exercise combined with MS of the muscles will positively affect self-rated recovery and mood, compared to exercise and sham-MS.

## Materials and Methods

### Design and Experimental Manipulation

The study was conducted according to a single-blind, randomized, placebo-controlled, and two-way crossover study. Participants performed a physical exercise twice on separate days and received microstimulation (MS) on 1 day and no microstimulation (sham-MS) on another day; half of the sample received MS on the first day and switched to sham-MS on the second test day, the other half received the treatment in reversed order. Before and after the exercise, and after MS, mood and levels of fatigue were assessed. The study was approved by the Ethics Review Committee Psychology and Neuroscience (reference number: 193_08_05_2018) at Maastricht University, Faculty of Psychology and Neuroscience.

### Participants

Participants were 20 healthy males aged in the range of 18–40 years with resistance training experience. They were recruited using advertisements and by word of mouth in the personal training studio of Bernd Stößlein in Kulmbach (Germany), where the testing took place.

#### Physical Exercise

The physical exercise consisted of conventional deadlifts, a reproducible exercise with a high transfer effect to everyday-life movements especially lifting heavy objects from the ground. The deadlift engages three large muscle groups of the knee, the hip, and the lower back (Farley, 1995). Deadlifts were performed using a straight 20 kg ATX® Bulls Bearing Bar-MK (Olympic Barbell) with a tensile strength of 206,000 pound-force per square inch on an ATX® Olympic Weightlifting Platform and ATX® HQ-Rubber Bumper Plates with possible weight increases of at least 0.5 kg (using Microplates/Friction Plates).

#### Study Procedures

Heart rate (HR) and systolic/diastolic blood pressure (BP) were measured pre-workout on both test days. Before the measurement, the BP cuff was placed on the participants’ left arm at heart level during sitting. After HR and BP were assessed, the first questionnaire was filled out.

Participants had to complete five sets of a six repetition maximum. One set entailed participants lifting the Barbell (with weights) from the ground, then lowering the bar with a time under tension (TUT) of 4,010, where 0 stands for no pause and 4 and 1, respectively, for the time (in seconds) the movement goes downwards and upwards. Thus, “4,010 TUT” means that the weight is lifted within 1 s to the top, followed by the lowering within 4 s to the ground, followed by the upward movement again, within pausing in between the upward-downward-upward movements. This had to be repeated six times, followed by a 120 s break; this was called a set.

The set was repeated five times in total. The six repetition maximum means that participants used as much weight as possible for maximally six repetitions. After the training, participants filled out the mood and recovery questionnaires for the second time, and they were attached to the microstimulation device for either microstimulation or sham treatment. After this, the mood and recovery questionnaires were filled out for the last time, and participants were asked to guess their condition (“treatment guess”).

#### Materials

**Figure 1:**
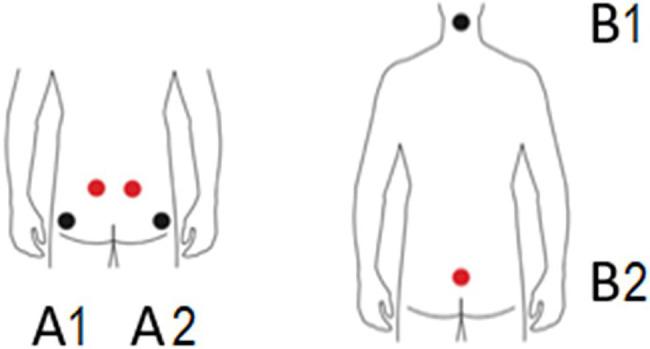
Positions of the electrodes. The picture was taken from JeeCee International NV with permission and adjusted. A1 and A2: upper and lower buttocks, B1: neck, and B2: sacrum.

The microstimulation (MS) was applied using the “B-E-S-t” device by JeeCee®. Adhesive patches were placed on the upper buttock (A1, blue cable, red electrode; A2, red cable, red electrode) and the lower buttock (A1, blue cable, black electrode; A2, red cable, black electrode), lower back (Sacrum, B1, yellow cable, red electrode), and the upper neck (B1, yellow cable, black electrode; see Figure 1). The cables’ color was of no importance for the treatment outcome; the purpose was to distinguish optically between the used channels and for more convenient and faster placement of the adhesive patches. In the MS and sham-MS conditions, the procedure was identical except for the actual stimulation, which was absent in the sham condition.

The frequency of the microcurrent ranges from 0.2 to 9999.9 Hz; the four channels (A1 and A2; B1 and B2) were available to use simultaneously. A session took 30 min, using three of the four channels, using the auto-treatment protocol for the gluteus maximus, gluteus medius, and gluteus minimus, based on the main muscles involved in deadlifts. The program’s frequencies were 0.3 Hz 200 μA (A1), 3.5 Hz 200 μA (A2), and 0.3 Hz 200 μA (B1) for 30 min and none at B2. The session was painless, apart from a slight tingling feeling that might be experienced.

A 3 M Littmann® Classic III stethoscope was used for evaluating blood pressure (mmHg). Heart rate was measured manually on the palpation point of the A. radialis, in beats per minute (bpm) on the radial forearm, about two fingers across the wrist using the index- and middle finger.

#### Demographics and Physiological Parameters

Participants’ age was asked, the vital signs (BP, HR) were taken, and body weight (kg) and length (m) were assessed. In addition, participants were asked about the consumption of medicinal and recreational drugs during the week preceding the experiment and whether caffeinated beverages were consumed on test days; if yes, how many cups. Lastly, participants were asked about their current diet (e.g., vegan, vegetarian, and meat-eater), their training schedule (number of workouts per week, the average number of hours per workout, and type of workout), lifting experience, and whether they had other remarks.

#### Mood Questionnaire

Five items to assess mood state were completed pre-workout, post-workout, and post-(sham)-MS using visual analogue scales (VAS). Each item (Well-rested, Happy, Sad, Sociable, and Exhausted) was self-rated on a 100 mm VAS with two anchors being “0” (not at all) and “10” (extremely; Watson et al., 1988).

#### Mental and Physical Recovery Questionnaire

The extent of physical exhaustion or recovery was assessed pre-workout, post-workout, and post-(sham)-MS using the Hecimovich-Peiffer-Harbough Exercise Exhaustion Scale (HPHEES; Hecimovich et al., 2014). The HPHEES consists of 14 VAS-items (100 mm); eight items ask how the person feels (recovered, energetic, refreshed, physically drained, mentally sharp, relaxed, mentally drained, and mentally clouded), four items about how easily the person can do something (walk, run, replicate their last game/event/competition, and train some more), and two items about physical sensations (how weak the person’s legs and/or arms feel and how much their muscles ache). This scale is intended to help quantify exercise-induced acute onset exhaustion, which was defined as loss of strength or vitality and decrease in physical activity (Hecimovich et al., 2014).

### Statistical Analysis

All questionnaire data entered the statistical program SPSS version 26.0. General linear model repeated measures analysis of variance (GLM RM ANOVA) was conducted including Treatment (2 levels, Sham-MS and MS) and Session (3 levels: Pre-workout (WO), Post-WO, and Post-Stim) as within-subject factors, and additionally Set (5 levels) for the weights that were lifted. Mood states data were checked for Pre-WO (“baseline”) differences between treatment conditions before entering GLM RM ANOVA using paired t-tests. Uncorrected (non-baseline corrected) data were used in the absence of differences.

In the case of non-sphericity, the Greenhouse-Geisser correction was applied. In the case of main effects, Bonferroni-corrected pairwise comparisons were conducted. The alpha criterion level of statistical significance for all analyses was set at *p* = 0.05. Partial eta squared (*η*2) was reported in case of significant effects in the ANOVA RM GLM to demonstrate the effect’s magnitude, where 0.01, 0.06, and 0.14 are defined as small, moderate, and large based on Cohen’s f, which represents small, medium, and large as, respectively, 0.10, 0.25, and 0.50 (Richardson, 2011). For the paired sample t-tests, Cohen’s *d* was reported where 0.2, 0.5, and 0.8 are defined as small, medium, and large effect sizes (Cohen, 2013).

## Results

### Demographic Information and Physiological Parameters

Twenty healthy male participants were included in the study; their mean (SD) age was 26.3 (SD = 4.83) years. The majority of the sample (95%, *N* = 19) was meat-eaters; one person was a vegetarian. They all had lifting experience with an average of 3.07 (*SD* = 0.83) training hours per week. All participants were microstimulation-naïve before the study. The participants’ average weight and length were 85.0 kg (1.9) and 1.82 m (0.1).

At the start of the session (Pre-WO), participants’ heart rate and blood pressure, and body weight and length were assessed. In both treatment conditions, paired sample t-tests did not reveal statistically significant differences between blood pressure (BP) and heart rate (HR). Systolic BP was on average (SE) 121.8 mmHg (0.8) in the Sham-MS condition and 122.4 mmHg (5.6) in the MS condition (*t*(19) = −0.73, *p* = 0.47); Diastolic BP was on average (SE) 76.7 mmHg (4.9) in the Sham-MS condition and 76.15 (5.33) in the MS condition (*t*(19) = 0.57; *p* = 0.58). HR was on average 68.3 (2.3) in the Sham-MS condition and 67.9 (2.8) in the MS condition (*t*(19) = 0.19, *p* = 0.85).

The majority (95%) did not use medicines in the week preceding the test day. Two participants took something on the days preceding the first test day; one took L-Thyrox (Sham-MS condition) and one took Lamotad (MS condition). There was no use of recreational drugs in the week preceding the test day. On test day 1, 50% did not consume caffeine; those who did drank on average 0.87 cups (*SD* = 1.05). On test day 2, 55% did not consume caffeine; those who did drank on average 0.75 cups (*SD* = 1.12). On average, there were 8 days between the two test days with a minimum of 2 days and a maximum of 35 days, which was the case for one participant.

### Workout Details

**Figure 2:**
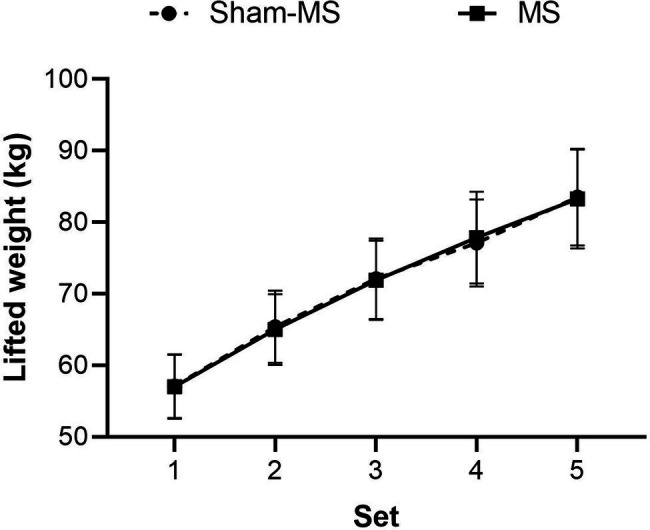
Mean (SE) of the weights (kg) that were lifted in the MS and Sham-MS condition.

The rest-time between sets was 120 s (SD = 0). The mean (SE) time between the end of the workout (WO) and the start of the treatment was 6.0 min (0.58) in the Sham-MS and 6.6 min (0.73) in the MS condition; this time between end-WO and the start of the treatment did not differ statistically as shown by a paired t-test (*t*(19) = −0.70, *p* = 0.49). Participants performed six repetitions in each of the five sets. GLM RM ANOVA revealed a main effect of Set (*F*_1.16_, _22.08_ = 68.41, *p* < 0.001, partial *η2* = 0.78) on the weight that was lifted, indicating that each subsequent set, participants lifted a larger weight than the set before (Figure 2). There was no main effect of Treatment (*F*_1,19_ = 0.001, *p* = 0.92, partial *η2* = 0.000) or Treatment by Set interaction effect (*F*_2.18, 41.34_ = 0.18, *p* = 0.85, partial *η2* = 0.009) on the lifted weight, indicating that there was no difference between treatment days in the weight that was lifted.

### Mood Questionnaire

**Figure 3:**
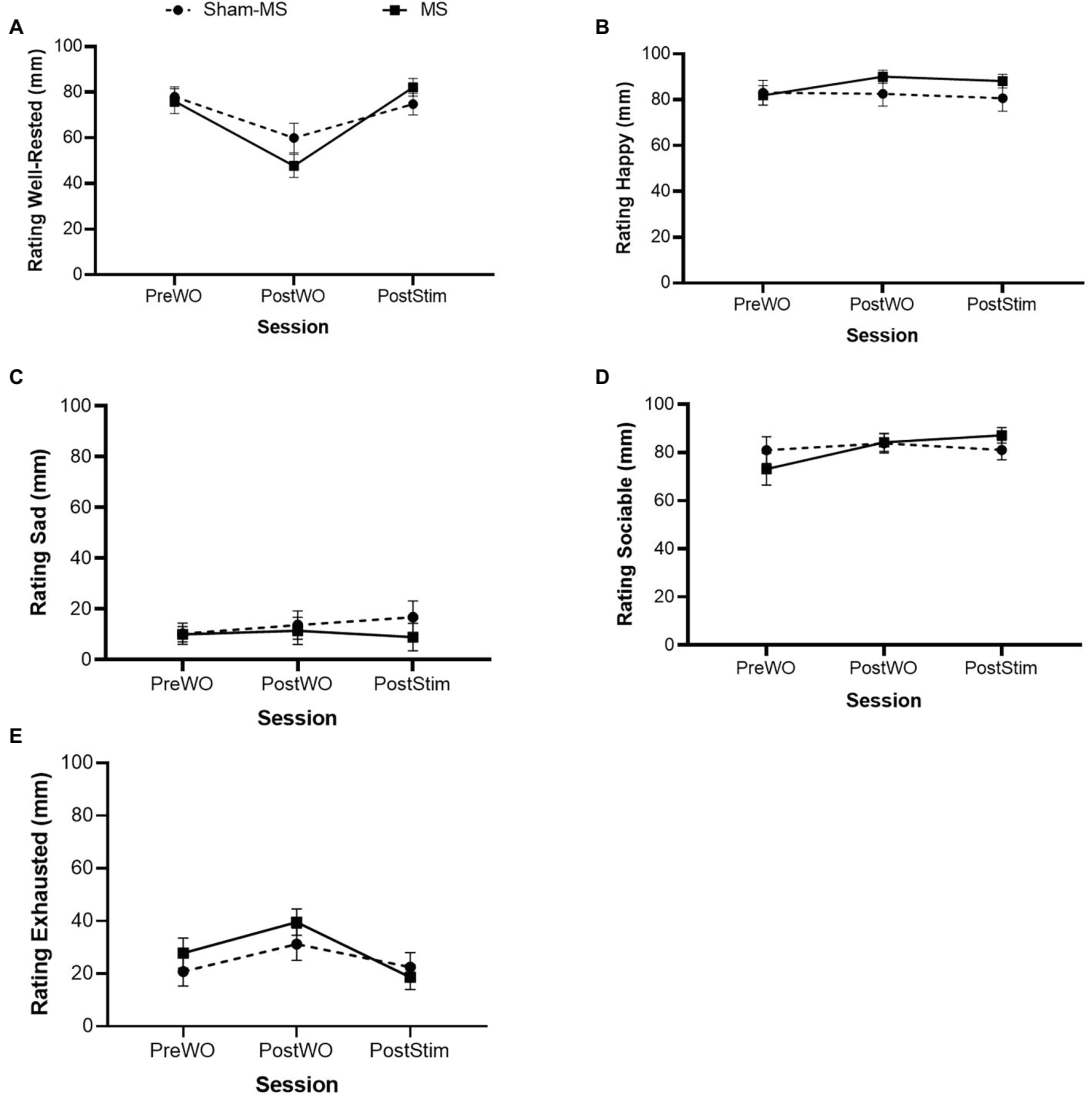
Self-rated mood states (mean and SE) in the Sham-MS and MS condition in the Pre-WO, Post-WO, and Post-Stim sessions. **(A)**: Well-rested, **(B)**: Happy, **(C)**: Sad, **(D)**: Sociable, **(E)**: Exhausted.

Paired sample *t*-tests did not reveal differences in mood between pre-WO in the Sham-MS and MS conditions (Table 1). Therefore, all data entered GLM RM ANOVA without “baseline” correction (Figure 3).

**Table 1:** Paired *t*-tests including Pre-WO (baseline) self-ratings of the mood state questions in the MS and sham-MS condition, and related values of *p* and effect sizes (d).

Self-rating	***t***(19)	***p***	***d***
Well-rested	0.348	0.732	0.08
Happy	0.35	0.817	0.05
Sad	0.088	0.931	0.02
Sociable	1.447	0.164	0.32
Exhausted	−0.858	0.401	0.19

#### Well-Rested

RM GLM ANOVA revealed a main effect of Session (*F*_1.55,29.42_ = 19.55; *p* < 0.001; partial *η2* = 0.51) and a Treatment by Session interaction (*F*_2,38_ = 5.21; *p* = 0.01; partial *η2* = 0.21) on Well-Rested. Regarding the former effect, Bonferroni-corrected pairwise comparisons revealed statistically significant differences between Pre-WO and Post-WO (mean difference: 23.20; *p* < 0.001), and between Post-WO and Post-Stim (mean difference: −24.64; *p* = <0.001). Self-ratings of well-rested went down from pre- to post-WO and went up from post-WO to post-stimulation. The ratings of well-rested decreased more in the MS condition after the WO and increased more post-stimulation than Sham-MS, suggesting that MS can enhance the feeling of well-rested after a WO. There was no main effect of treatment on well-rested (*F*_1,19_ = 0.27; *p* = 0.61; partial *η2* = 0.01).

#### Happy

RM GLM ANOVA did not reveal main effects of Treatment (*F*_1, 19_ = 1.22; *p* = 0.28; partial *η2* = 0.06) or Session (*F*_1.38, 26.25_ = 0.52; *p* = 0.53; partial *η2* = 0.03) or an interaction effect of Treatment by Session (*F*_1.21, 23.04_ = 1.87; *p* = 0.18; partial *η2* = 0.09) on self-ratings of Happy. Findings suggest that neither the workout or the MS influenced ratings of happiness.

#### Sad

RM GLM ANOVA did not reveal main effects of Treatment (*F*_1, 19_ = 0.34; *p* = 0.56; partial *η2* = 0.02) or Session (*F*_1.49, 28.37_ = 0.53; *p* = 0.54; partial *η2* = 0.03) or an interaction effect of Treatment by Session (F_1.33, 25.37_ = 0.78; *p* = 0.42; partial *η2* = 0.039) on self-ratings of Sad. Findings suggest that neither the WO or the MS influenced ratings of sadness.

#### Sociable

RM GLM ANOVA revealed a main effect of Session (*F*_1.28, 24.24_ = 4.13; *p* = 0.03; partial *η2* = 0.20) and a Treatment by Session effect (*F*_1.30, 24.78_ = 4.68; *p* = 0.03; partial *η2* = 0.20) on self-ratings of Sociable. Regarding the former effect, Bonferroni-corrected pairwise comparisons showed that self-ratings of Sociable went down from Pre-WO to Post-WO (mean difference = −6.985; *p* = 0.043). Sociable’s self-ratings increased over the sessions in the MS condition compared to Sham-MS with the highest rating after MS. The ratings in the Sham-MS condition went down at the last session, suggesting that MS contributed to increased feelings of sociableness after a WO. Analysis did not show a main effect of Treatment (*F*_1, 19_ = 0.03; *p* = 0.86; partial *η2* < 0.01) on ratings of sociableness.

#### Exhausted

RM GLM ANOVA revealed a main effect of Session (*F*_2,38_ = 4.786; *p* = 0.014; partial *η2* = 0.201) on self-ratings of Exhausted. Bonferroni-corrected pairwise comparisons showed ratings decreased from Post-WO to Post-stimulation (mean difference = 17.794; *p* = 0.014). Analyses did not show a main effect of Treatment (*F*_1,19_ = 0.75; *p* = 0.40; partial *η2* = 0.04) or an interaction effect of Treatment by Session (*F*_2,38_ = 0.86; *p* = 0.43; partial *η2* = 0.04) on self-ratings of Exhausted.

### Mental and Physical Recovery Questionnaire

Paired sample *t*-tests including the pre-WO ratings of the positive and negative items in the two treatment conditions showed an absence of baseline differences for the positive items (*t*(19) = 0.78; *p* = 0.45, *d* = 0.2), while there was a statistically significant baseline difference for the negative items (*t*(19) = −2.23; *p* = 0.04; *d* = 0.5). Therefore, baseline-corrected ratings of negative items entered the GLM RM ANOVA.

#### Positive Items HPHEES

**Figure 4:**
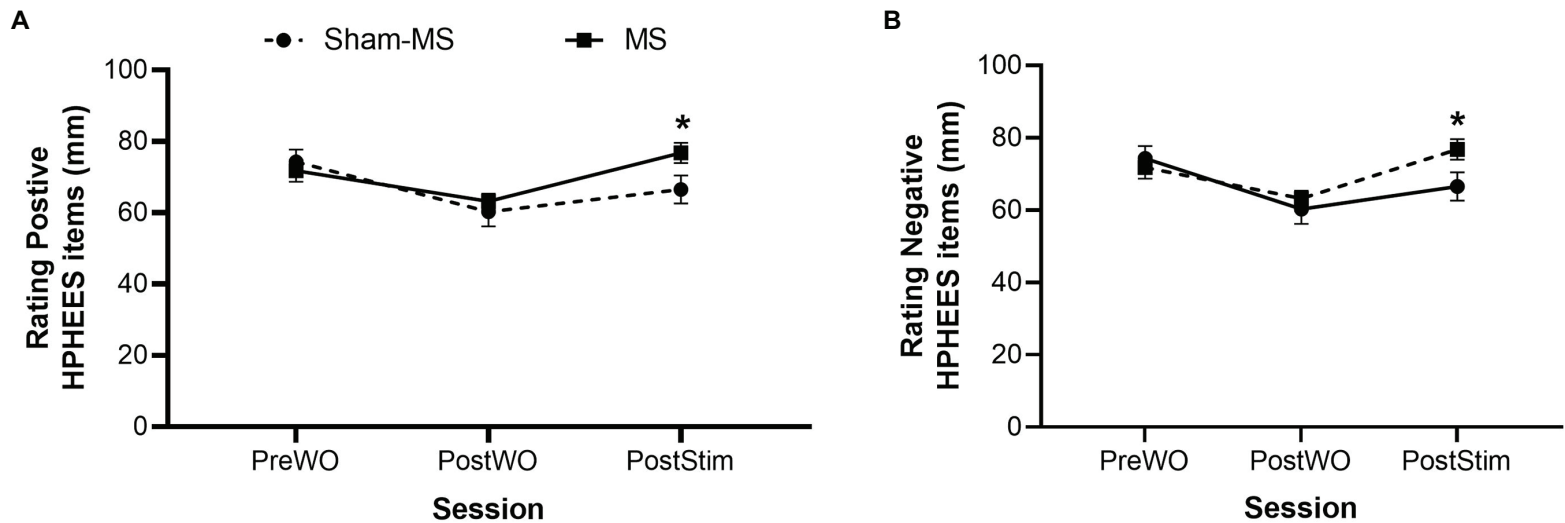
Self-ratings (mean and SE) of the positive **(A)** and negative **(B)** items of the HPHEES after MS and Sham-MS. *Signifies statistical significance.

GLM RM ANOVA revealed a main Session effect (*F*_2,38_ = 13.64, *p <* 0.001, partial *η2* = 0.42) on the total score of the positive items of the HPHEES. Bonferroni-corrected pairwise comparisons showed that the self-rating of the positive items (e.g., “I feel recovered, I could easily walk”) decreased from Pre-WO to Post-WO (mean difference: 11.25; *p* = 0.002) but increased from Post-WO to Post-Stimulation (mean difference: 9.88, *p* < 0.001). The Treatment by Session interaction effect (*F*_2,38_ = 10.56, *p* < 0.001, partial *η2* = 0.36) indicated that the increase in self-ratings of the positive items was larger when participants received MS compared with Sham-MS. This suggests that exercise-induced exhaustion can be diminished with microstimulation of the muscles. Analysis did not reveal a main effect of Treatment (*F*_1,19_ = 1.47, *p* = 0.24, partial *η2* = 0.07; Figure 4A).

#### Negative Items HPHEES

GLM RM ANOVA revealed a main effect of Treatment (*F*_1,19_ = 30.70, *p* < 0.001, partial *η2* = 0.62), Session (*F*_1,19_ = 10.58, *p* = 0.004, partial *η2* = 0.36), and a Treatment by Session interaction effect (*F*_1,19_ = 5.79, *p* = 0.03, partial *η2* = 0.23). The self-ratings of negative HPHEES items (e.g., “physically drained, weak arms and legs”) were reduced from Post-WO to Post-Stimulation, and this effect was more pronounced when participants received MS compared with Sham-MS (Figure 4B).

### Treatment Guess

In 60% of the cases (*N* = 24), participants correctly guessed the treatment condition. On 16 occasions, they guessed incorrectly; 56% of those incorrect guesses (*N* = 9) were made on day 1, 44% (*s* = 7) on day 2. Sham-MS was mistaken for MS on 50% of the occasions, so at chance level, MS was mistaken for Sham-MS on 30% of the occasions, suggesting that MS produces changes observed by some.

## Discussion

The present study aimed to test the effects of physical exercise and microstimulation (MS) of the muscles (gluteus maximus, gluteus medius, and gluteus minimus) on self-rated mood and physical and mental recovery. Based on previous work, it was hypothesized that physical exercise followed by MS of the muscles would positively affect self-rated recovery and mood, compared to exercise followed by sham-MS. Findings were partially in line with the hypothesis; MS increased the self-ratings of being well-rested and sociable, and, most importantly, reduced the feeling of exercise-induced exhaustion. There were no MS-dependent effects on ratings of feeling sad, happy, or exhausted. However, the workout, independent of MS, negatively influenced the feeling of exhaustion, which is expected after a workout.

Previous research demonstrated that MS at the Shenmen acupoint (HT7) changed brain activity as measured with EEG, indicating decreased sleepiness and increased positive mood in healthy volunteers (Cheung et al., 2015). The present study’s findings partially align with Cheung et al. (2015), as participants felt more well-rested and less exhausted by the exercise after MS. Something similar was recently demonstrated by Piras et al. (2021) who showed speeded physical recovery when RT was followed by MS (Piras et al., 2021). However, unlike Cheung et al. (2015), no effects of MS on positive mood or mood in general were found in the present study. The methodological differences in both studies concerning stimulation location and duration might explain the differential effect pattern; whereas Cheung et al. (2015) stimulated for 10 min at an acupuncture point, the stimulation duration in the present study was three times longer at muscle attachment points. Comparative studies are needed to investigate whether stimulation characteristics differentially influence mood or whether there are additional variables like the extent of feeling well-rested or recovered explaining this.

Next to the abovementioned effects, the positive effect of MS on sociability after physical exercise is an interesting finding, adding to the favorable effect pattern of MS in specific populations, and warranting further research. It has been suggested that MS affects energy and influences communication systems in the body (Smith, 2002), indicating that it can be an effective treatment for various physical and psychological problems. Additionally, it is known that physical (aerobic) exercises, because of a reduction of cardiovascular risk factors, are an effective means when dealing with cognitive problems, which can be more prominent in the elderly (Kirk-Sanchez and McGough, 2014), but also in psychological conditions like depression, where aerobic as well as anaerobic exercises can be part of the therapeutic mechanism (Doyne et al., 1987). When the populations above are treated with MS after having exercised, they might be more willing to engage in exercises because of the effects on sociability, next to the abovementioned positive effects, which might, in its turn, enhance their mental condition and overall wellbeing.

While the present study had its strengths, it also came with limitations, both will be addressed here. First, the participants were asked to guess the treatment condition at the end of each session. Findings showed that while guesses for sham-MS were at chance level, indicating that participants were unsure whether or not they received MS, when having received sham-MS; more people correctly guessed that they received MS. This partial treatment unblinding could have been caused by the subtle sensations that can be experienced during MS. However, MS affected a selection of parameters (e.g., sociability, exercise-induced exhaustion, and well-rested), and not all (e.g., mood states) indicate that the effect is valid and not caused by unblinding and socially desirable behavior toward study outcomes. Nonetheless, future studies might want to include a treatment guess question to consider the biases above. Another related limitation is that the study was single-blind; the experimenter knew the treatment condition. Also here, the argument that the effects of MS were selective speaks in favor of the lack of confounding by this methodological choice. The study’s strengths were using a within-subject design where participants are their own control to reduce variance and the inclusion of the sham-MS condition.

While recovery after exercise is an important topic for professionals and nonprofessionals who sport, microstimulation might be an additional strategy to the existing range (Peake, 2019), or it might even be combined with nutritional, physical, and sleep interventions. It might also be a non-invasive, cost-effective, and safer option for muscle regeneration than intermittent steroid use (Quattrocelli et al., 2017). To optimize MS after a WO, we need to understand what factors can influence the strength of the effect. Future research might want to take physiological parameters like body temperature and hydration into account and assess parameters of tissue damage pre- and post-WO and post-stimulation. Additionally, the sample will have to be diversified, including both males and females, and the type of exercise, for example, strength and endurance training, and combinations thereof (Eklund et al., 2016), and the location, duration, and frequency of stimulation has to be varied and linked to the duration of effects.

The present findings can have important implications for professional sporters and nonprofessionals trying to get the most out of their workout and afterward. Future studies using a double-blind approach including different types of exercises, different durations of programs, and both sexes can shed more light on the full potential of microstimulation on mood and mental and physical recovery in the post-workout phase.

## Data Availability Statement

The raw data supporting the conclusions of this article will be made available by the authors, without undue reservation.

## Ethics Statement

The studies involving human participants were reviewed and approved by the Ethics Review Committee Psychology and Neuroscience (reference number: 193_08_05_2018) at Maastricht University, Faculty of Psychology and Neuroscience. The participants provided their written informed consent to participate in this study.

## Author Contributions

BS and KK designed the study and wrote the manuscript. BS collected the data. KK performed the statistical analyses. All authors contributed to the article and approved the submitted version.

## Conflict of Interest

BS is employed by Bernd Stößlein Personal Training (BSPT)-Studio. He received adhesive patches from JeeCee to use in the study.

The remaining author declares that the research was conducted in the absence of any commercial or financial relationships that could be construed as a potential conflict of interest.

## Publisher’s Note

All claims expressed in this article are solely those of the authors and do not necessarily represent those of their affiliated organizations, or those of the publisher, the editors and the reviewers. Any product that may be evaluated in this article, or claim that may be made by its manufacturer, is not guaranteed or endorsed by the publisher.
