# Corrigendum: Self-rated recovery and mood before and after resistance training and muscle microcurrent application

**DOI:** 10.3389/fpsyg.2024.1544656

**Published:** 2025-01-20

**Authors:** Bernd A. C. Stößlein, Kim P. C. Kuypers

**Affiliations:** Department of Neuropsychology and Psychopharmacology, Faculty of Psychology and Neuroscience, Maastricht University, Maastricht, Netherlands

**Keywords:** frequency-specific microcurrent, microstimulation, deadlifts, resistance training, self-rated recovery, mood

In the published article, there was an error in the **Conflict of Interest**. The statement was missing a disclosure for Author Bernd A. C. Stößlein.

The original Conflict of Interest previously stated:

“The authors declare that the research was conducted in the absence of any commercial or financial relationships that could be construed as a potential conflict of interest.”

The corrected statement appears below:

